# No difference in learning outcomes and usability between using controllers and hand tracking during a virtual reality endotracheal intubation training for medical students in Thailand

**DOI:** 10.3352/jeehp.2021.18.22

**Published:** 2021-08-18

**Authors:** Chaowanan Khundam, Naparat Sukkriang, Frédéric Noël

**Affiliations:** 1 Informatics Innovative Center of Excellence (IICE), School of Informatics, Walailak University, Nakhon Si Thammarat, Thailand; 2 School of Medicine, Walailak University, Nakhon Si Thammarat, Thailand; 3 Institute of Engineering, Université Grenoble Alpes, Grenoble, France; Hallym University, Korea

**Keywords:** Endotracheal intubation, Learning, Medical education, Personal satisfaction, Virtual reality

## Abstract

**Purpose:**

We developed a virtual reality (VR) endotracheal intubation training that applied 2 interaction modalities (hand-tracking or controllers). It aimed to investigate the differences in usability between using hand tracking and controllers during the VR intervention for intubation training for medical students from February 2021 to March 2021 in Thailand.

**Methods:**

Forty-five participants were divided into 3 groups: video only, video with VR controller training, and video with VR hand tracking training. Pre-test, post-test, and practice scores were used to assess learning outcomes. The System Usability Scale (SUS) and User Satisfaction Evaluation Questionnaire (USEQ) questionnaires were used to evaluate the differences between the VR groups. The sample comprised 45 medical students (undergraduate) who were taking part in clinical training at Walailak University in Thailand.

**Results:**

The overall learning outcomes of both VR groups were better than those of the video group. The post-test scores (P=0.581) and practice scores (P=0.168) of both VR groups were not significantly different. Similarly, no significant between-group differences were found in the SUS scores (P=0.588) or in any aspects of the USEQ scores.

**Conclusion:**

VR enhanced medical training. Interactions using hand tracking or controllers were not significantly different in terms of the outcomes measured in this study. The results and interviews provided a better understanding of support learning and training, which will be further improved and developed to create a self-learning VR medical training system in the future.

## Introduction

### Background/rationale

Virtual reality (VR) is commonly used to enhance practitioners’ learning and engagement. Learning by doing is the most effective method to understand lessons; however, it is not feasible to make repeated mistakes in practice for some techniques due to risks or resource constraints. Education and training using VR can provide content in a mixed-media format based on creating a virtual world, wherein users see and interact with their surroundings virtual environment in 3 dimensions. Learners can try to perform a technique by themselves as long as they want and may make mistakes without any negative effects, allowing them to practice repeatedly and learn from their mistakes. These capabilities of VR have direct implications for medical education and training [1]. VR training provides realistic learning experiences, supporting learners who are inspired to discover the material on their own. Learners can explore a lesson’s content to see how its elements work and are more likely to engage in the lesson experientially. Moreover, VR provides an opportunity to learn by interacting with lessons instead of passively reading or listening to experts. The widespread use of simulation training is based on patient safety concerns, focusing on improving the quality of medical services and clinical outcomes. Several studies have shown that learning in VR simulations was more effective than traditional clinical training [2-4]. It has been reported that practice using VR simulations improved medical students’ learning in many domains [5].

Intubation is a standard procedure of respiratory care for patients. However, learning and practicing intubation is a risky and challenging process for beginners, because intubation involves manipulating patients’ airways using a laryngoscope and inserting an endotracheal tube into the trachea. Practicing with patients can lead to life-threatening risks when performed by inexperienced operators. Furthermore, the specific steps of intubation involve important differences in the details of the procedure. Supervisors should focus on guiding various sub-behaviors to help trainees improve their skills. Practice usually takes place using manikins; however, equipment is often insufficient for individual learners and manikins require expert guidance to use.

For these reasons, training with VR simulations is beneficial for encouraging and supporting learners as they become proficient in their skills. However, there are various forms of VR training. Interactions through controllers are at the foundation of VR [6], and VR headsets currently offer controllers as an essential accessory without additional purchases. Therefore, many VR applications have user interactions with the environment through controllers, which allow users to perform actions such as touching objects and manipulating objects according to their hand position and button commands. Another mechanism for controlling VR applications is the use of hand tracking for interactions. Hand tracking is a new technology in VR that uses a built-in camera on the VR headset to detect a user’s position and hand gestures. Therefore, VR applications can use hand tracking to control object touching and manipulation by detecting the user’s hand, similar to what is possible using controllers [7].

### Objectives

**Fig. 1.**
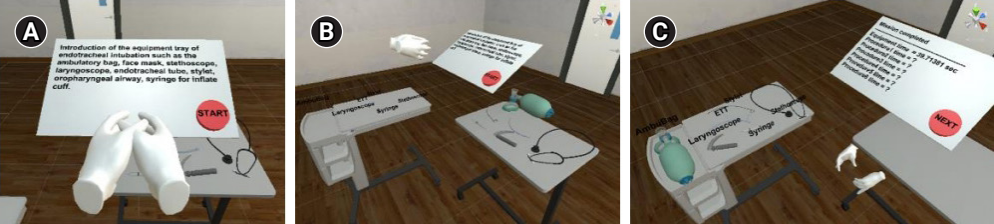
Introductory scene where the user has to pick up and drop each tool according to its name: (A) instruction before starting; (B) overall virtual environment; and (C) conclusion after finishing the introductory scene.

**Fig. 2.**
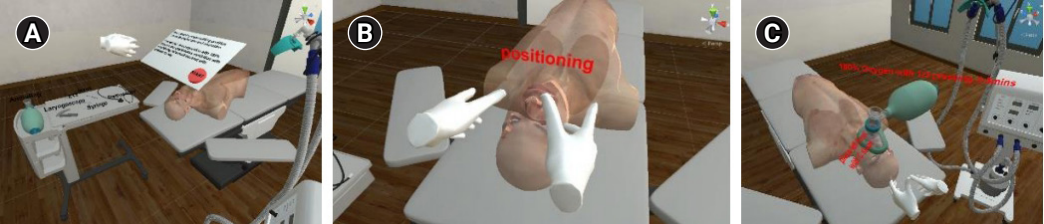
Scenes from procedures 1 and 2, where the user is required to position the virtual patient and pre-oxygenation: (A) overall virtual environment; (B) sniffing position; and (C) pre-oxygenation with an ambulatory bag.

**Fig. 3.**
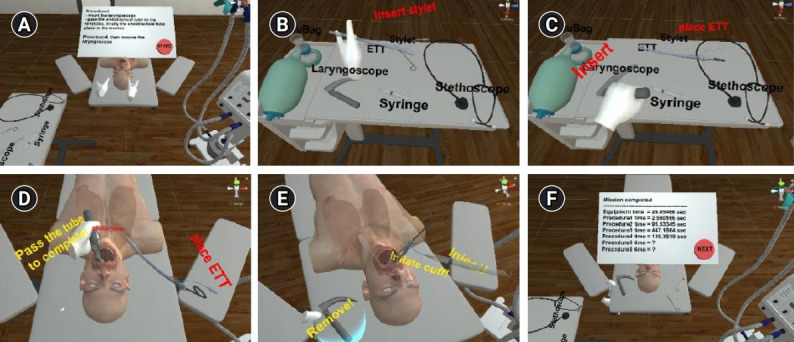
Scenes from procedures 3 and 4 where the user is required to insert the laryngoscope and pass the endotracheal tube, and then inflate the cuff to complete the ask: (A) overall virtual environment and instruction; (B) stylet insertion; (C) procedure guide; (D) passing the endotracheal tube; (E) inflating the cuff; and (F) time summary.

**Fig. 4.**
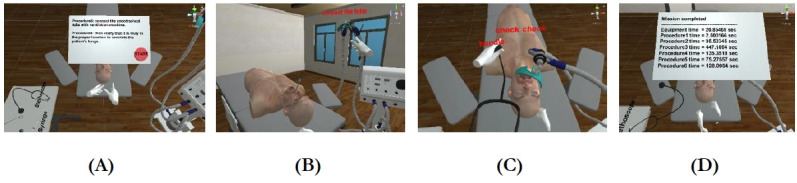
Scenes from procedures 5 and 6 where the user is required to connect the ventilation machine and verify the endotracheal tube position: (A) overall virtual environment and instruction; (B) connecting the ventilation machine; (C) verification; and (D) time summary.

**Fig. 5.**
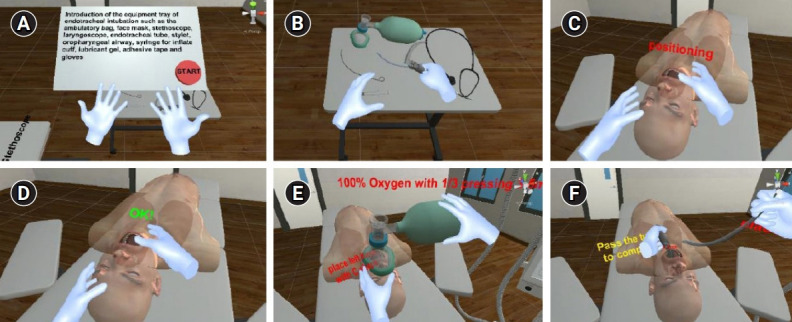
The virtual intubation training with virtual reality hand tracking; all procedures are the same, but the virtual hands show the user’s real hand gestures: (A) the start button can be touched using a virtual hand; (B) hand grab for selection; (C) hand collision for positioning; (D) result of hand collision; (E) squeezing the hand; and (F) controlling the hand.

**Fig. 6.**
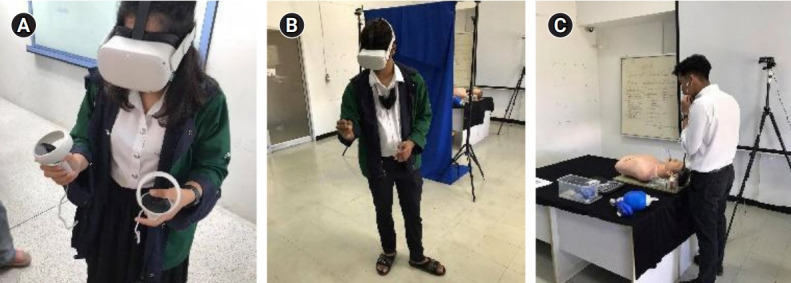
Experiment using virtual reality (VR) training: (A) VR controllers (group 2); (B) VR hand tracking (group 3); and (C) practice session.

**Fig. 7.**
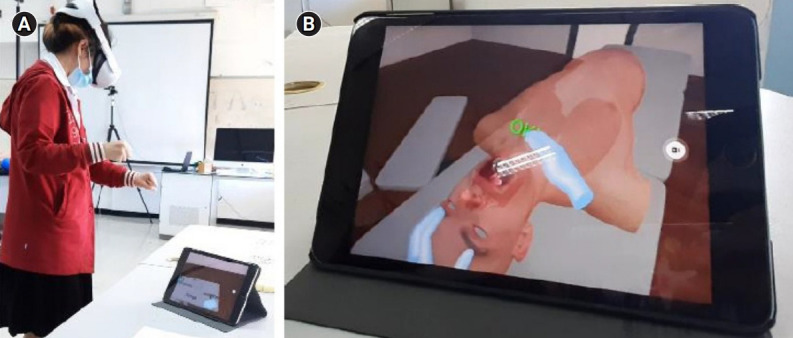
Virtual reality connection during experiment: (A) participant’s interaction; and (B) Oculus Quest display cast to an iPad screen in real-time.

The present study aimed to investigate the differences in VR intubation training as a case study to explore the differences between using controllers (Figs. 1–4) and hand tracking (Fig. 5) for learning in VR (Figs. 6, 7). The following variables were compared between controller interaction and hand tracking: learning outcomes, practice scores, usability, ease of use, and satisfaction with the VR system. Feedback from real users can provide useful input for understanding the design and development of VR applications to make them more effective from a functional standpoint for learning and practice in medical training. The research question was as follows: are there any differences in learning outcomes and usability between using controllers and hand tracking in VR medical training?

## Methods

### Ethics statement

This study was approved by the Ethics Committee of Human Rights Related to Research Involving Human Subjects, Walailak University, Thailand (WUEC-20-031-01). Informed consent was obtained from subjects.

### Study design

This was a pre- and post-intervention comparative observational study, involving a comparison of 3 groups and interviews.

### Setting

We conducted this cross-sectional study at Building B6 in the Laboratory Teaching Center of the School of Medicine, Walailak University, Nakhon Si Thammarat, Thailand, from February 17, 2021, to March 5, 2021. The interventional program is available in Supplement 1. The research protocol and experimental process for each group are presented in Table 1 and Dataset 1. None of the participants had previously studied intubation. We divided them into 3 groups, each of which contained 15 students who learned intubation in a different environment. Group 1 consisted of 15 students who received only video learning (Supplement 2). Group 2 consisted of 15 students who received both video learning and VR training with controller interactions (Supplement 3). Group 3 consisted of 15 students who received video learning and VR training with hand tracking interactions (Supplement 4).

### Participants

The sample comprised participants who were 3rd-year medical students (undergraduate) taking part in clinical training at Walailak University, Nakhon Si Thammarat, Thailand. Total number of students was 48. The researchers included only data from students who completed all tasks, including the pre- and post-intervention questionnaires and practice tests (n=45), and no data were excluded for this reason. The participants all voluntarily participated in the study after providing informed consent.

### Variables

The following variables were analyzed: pre-test and post-test scores for learning outcomes; usability for the System Usability Scale (SUS) tool; usefulness, ease of use, ease of learning, and satisfaction for the User Satisfaction Evaluation Questionnaire (USEQ) tool; and emotional, instrumental, and motivational experiences in the interviews.

### Data sources/measurement

Our experiment investigated learning outcomes and usability. Learning outcomes were assessed in 2 parts. In the first part on knowledge and understanding, pre- and post-test scores were assessed using 10 questions testing intubation knowledge with a total score of 10 (Supplement 5). The reliability of the test was pre-tested with a non-study sample using the Cronbach α coefficient. The second part related to the practice assessment (Supplement 6). The validity of the research instruments for evaluating the success of learning was checked by 3 experts to assess students’ ability to practice with the manikin. The item-objective congruence index was used to determine content validity. There were 7 steps to be followed with a total score of 14. On both VR applications, usability was assessed using the SUS [8] and USEQ [9], which are 5-point Likert-scale questionnaires. The SUS was used to evaluate the usability of the VR application, while the USEQ was used to assess its usefulness, ease of use, ease of learning, and satisfaction.

### Bias

The researchers explained which protocol would be followed for each group, and each student then voluntarily chose a group according to his or her preferences. It may be possible that less self-motivated learners might not opt to try a new modality.

### Study size

The sample size was chosen (45 out of a total of 48 medical students) as a convenient sample for this study. These students voluntarily participated in this study and enrolled into one of 3 groups.

### Statistical methods

Table 2 shows the means and standard derivations of the pre-test, post-test, and practice scores. The results of normality testing for all 3 groups showed that the pre-test, post-test, and practice scores had a normal distribution. One-way analysis of variance (ANOVA) was thus used as a statistical model to analyze the differences among these groups. Additionally, the SUS scores of both VR groups also exhibited a normal distribution. Therefore, the independent t-test was used to examine the differences between the VR groups. However, the usefulness, ease of use, ease of learning, and satisfaction of both VR groups showed non-parametric distributions; for this reason, the Mann-Whitney test was used to analyze the differences in those scores between the VR groups.

## Results

### Pre-test scores

As shown in Table 3, the pre-test scores of all groups were not significantly different (P=0.468), implying that all 3 groups had comparable prior knowledge (Dataset 2).

### Post-test scores

As presented in Table 3, the post-test scores of all groups were significantly different (P=0.028) (Dataset 3). When using the ANOVA post hoc test (least significant difference, LSD), we found the following pairwise P-values: video versus VR controllers, P=0.012; video versus VR hand tracking, P=0.043; and VR controllers versus VR hand tracking, P=0.581. This result shows that the video group had significantly lower post-test scores than both VR groups. However, the post-test scores of both VR groups were not significantly different. Therefore, the post-test evaluation indicated that participants who learned with VR had a higher level of knowledge than participants who only watched a video.

### Practice scores

As shown in Table 3, the practice scores of all groups were different with a high level of significance (P<0.001, Dataset 4). On the practice test, we found that participants who learned with VR had different scores from those of participants who only watched the video. Moreover, the average score on the practice test of participants who learned with VR was higher than that of participants who only watched the video. The ANOVA post hoc test (LSD) showed that the practice scores were not significantly different between the VR controller and VR hand tracking groups (P=0.168).

### VR usability results between using controllers and hand tracking

The SUS score was 67.17 (almost satisfactory) for the VR controllers and 60.17 (poor) for VR hand tracking. As shown in Table 4, these scores were not statistically significant, consistent with the interviews finding that there were areas for improvement in both VR applications (Dataset 5).

Similarly, as shown in Table 5, the usefulness, ease of use, ease of learning, and satisfaction scores were not significantly different between both VR applications. Therefore, to summarize, the experiment of VR medical training on intubation did not show a significant difference in usability according to the use of controllers or hand tracking.

### Interview results

The results from the interviews with 30 students in the 2 VR trial groups were broadly consistent, with the following details:

#### Emotional experiences

Participants gave positive comments such as: “Intubation training with a VR application is easy to learn,” “It was fun, just like playing a game,” and “It was like doing it in a real situation.” Some of them gave feedback to improve our VR application: “There should be a guide telling me whether the steps are right or wrong to make it easier to understand.” However, 2 of the 30 participants felt that watching videos was easier to understand than using VR.

#### Instrumental experiences

Participants gave positive comments such as: “3D rendering makes it easier to understand” and “Touching, grabbing, and moving hands make understanding easier than learning from the video.” Some feedback was given for improvements: “The system automatically rotates the device, instead of doing it manually” and “Its usage is not the same as the real device.” Three of the 30 participants felt that interacting through VR did not improve their understanding.

#### Motivational experiences

Twenty-nine participants recommended others to use this VR training application because it was easy to understand, fun to learn, something new to try, and was an authentic learning experience. Only 1 participant did not recommend it, because it was not yet practical for users to implement the program.

When asked what features users would like to add to this VR application to make intubation training better, the answers were as follows: “Do not let objects pass through the virtual manikin,” “I want a voice to tell me what was done wrong,” “I want a system with force feedback,” “I want a response at the end of each procedure such as good or excellent to simulate e playing games,” “There needs to be a more detailed description,” “I do not want the auto-snap function, I want the system that notifies me if the operation is right or wrong,” and “I want a time-keeper that resembles the real situation.”

## Discussion

### Key results

Although no significant differences were found between both VR groups, the results for the VR controller group were slightly better. The use of hand tracking technology is not perfect and there remains room for improvement of its accuracy. The detection of hand gestures is sometimes not stable. Handling and dropping still have some delays at times, resulting in a lower usability score on the USEQ. However, this issue related to usability did not affect learning outcomes. We found from the interviews that users who gave a low usability score would not recommend the VR system to others, but did give comments on how to improve it.

### Interpretation

The video-only group’s average post-test and practice scores were less than those of the groups who used VR simulations. Focusing on the interactions in VR intubation training, we found that there was no significant difference according to the use of controllers or hand tracking to interact with virtual objects in the simulations. In particular, the post-test and practice scores of both VR groups, as indicators of learning outcomes, were not meaningfully different. We found that both VR groups’ SUS and USEQ scores, as measures of VR usability, were also not significantly different. However, the average SUS score of the VR controller group was slightly higher than that of the VR hand tracking group. This is consistent with the findings of the interviews that users were better at push-button interactions than at hand gestures. The interviews suggested that the participants wanted to add learning assistance systems such as sound systems, validation systems, or force feedback systems to make it easier for them to learn by themselves.

### Comparison with previous studies

Our results on learning outcomes from the case study of intubation training using VR are consistent with the findings of other studies that VR contributes to learning and practice. Simulation training was associated to improve knowledge and skill outcomes [2,3] as well as practice performance [4,5].

### Limitations

Some of the equipment used for training in the VR application might have inconsistent appearances, leading to confusion among some students during the practice test.

### Conclusion

The learning outcomes from the post-test and practice scores indicated that the 2 VR training sessions had comparable outcomes. The usability scores for all categories revealed that utilizing a VR application was similar with either controllers or hand tracking. Based on our research question, we conclude that using controllers or hand tracking for the case study of intubation training in VR made no difference in terms of learning results and usability. A future task is to develop VR applications that enable user-suggested functionalities and examine the factors that contribute to better learning results and interaction usability.

## Figures and Tables

**Table 1.** The research protocol and experiment process of each group according to the VR application

Time (min)	Group 1 (control)	Group 2 (VR with controllers)	Group 3 (VR with hand tracking)
5–10	Take the pre-test to test prior knowledge about intubation.	Take the pre-test to test prior knowledge about intubation.	Take the pre-test to test prior knowledge about intubation.
6	Study intubation from the video.	Study intubation from the video.	Study intubation from the video.
10–15	-	Practice by self-studying using VR application with interaction through controllers.	Practice by self-studying using the VR application with interaction through hand tracking.
5–10	Take the post-test to evaluate intubation knowledge after learning.	Take the post-test to evaluate intubation knowledge after learning.	Take the post-test to evaluate intubation knowledge after learning.
5–10	Intubation practice test with a manikin.	Intubation practice test with a manikin.	Intubation practice test with a manikin.
5	-	Questionnaires and interviews about the VR experience.	Questionnaires and interviews about the VR experience.

VR, virtual reality.

**Table 2.** Pre-test, post-test, and practice scores of all experimental groups

Group	Pre-test (n=10)	Post-test (n=10)	Practice (n=14)
Video	4.40±1.88	6.47±1.45	6.42±2.29
VR controller	4.07±1.33	7.73±1.22	10.62±1.90
VR hand tracking	3.67±1.59	7.47±1.25	11.42±2.11

Values are presented as mean±standard deviation. VR, virtual reality.

**Table 3.** The analysis of variance table for pre-test, post-test, and practice scores

Source of variation	SS	MS	F	Sig.
Pre-test	4.044	2.022	0.773	0.468
Post-test	13.378	6.689	3.880	0.028^a)^
Practice	216.400	108.200	44.462	0.000^b)^

SS, sum of squares; MS, mean square; Sig., significance.

a) Significant at P<0.05.

b) Highly significant at P<0.01.

**Table 4.** Independence sample t-test results of System Usability Scale score for VR training

Group	No.	Mean	SD	SE	t-value	Sig.
VR controllers	15	67.1667	18.91869	2.022	0.849	0.588
VR hand tracking	15	60.1667	25.71247	108.200		

VR, virtual reality; SD, standard deviation; SE, standard error; Sig., significance.

**Table 5.** Mann-Whitney U-test results for learning, training, ease of use, and satisfaction scores for VR training according to the use of controllers or hand tracking

Usability scale	Group	No.	Mean ranks	Sum ranks	U-value	P-value
Usefulness	VR controllers	15	16.03	240.5	104.5	0.75656
	VR hand tracking	15	14.97	224.5		
Ease of use	VR controllers	15	16.3	244.5	100.5	0.63122
	VR hand tracking	15	14.7	220.5		
Ease of learning	VR controllers	15	16	240	105	0.77182
	VR hand tracking	15	15	225		
Satisfaction	VR controllers	15	17.03	255.5	59.5	0.35238
	VR hand tracking	15	13.97	209.5		

VR, virtual reality.

## Footnotes

**Data availability**
Data files are available from Harvard Dataverse: https://doi.org/10.7910/DVN/5TXL3N Dataset 1. Student list and schedule.

Dataset 2. Pre-test scores of all groups.

Dataset 3. Post-test scores of all groups.

Dataset 4. Practice scores of all groups.

Dataset 5. System Usability Scale (SUS) and User Satisfaction Evaluation Questionnaire (USEQ) questionnaires scores of virtual realitygroups.

## Supplementary materials

Data files are available from Harvard Dataverse: https://doi.org/10.7910/DVN/5TXL3N

Supplement 1. Design and development of training.

Supplement 2. Intubation video.

Supplement 3. Tutorial video for virtual reality controller training.

Supplement 4. Tutorial video for virtual reality hand tracking training.

Supplement 5. Pre- and post-tests items for the knowledge test of the intubation.

Supplement 6. Full questionnaire distributed to the cardiology residents.

Supplement 7. Audio recording of the abstract.
