# Supplementary material for: No difference in learning outcomes and usability between using controllers and hand tracking during a virtual reality endotracheal intubation training for medical students in Thailand
Source: J Educ Eval Health Prof. 2021 Aug 18;18:22. doi: 10.3352/jeehp.2021.18.22 (PMC8616725; doi:10.3352/jeehp.2021.18.22)
Supplement: Supplementary file 6 — Supplement 1. Design and development of training. [file jeehp-18-22-suppl1.docx]

**Supplement 1.** Design and development of training

This research requires a virtual reality (VR) headset that supports inside-out technology and hand tracking. The Oculus Quest with Touch controllers supports both technologies, an appropriate tool to use in the experiment. The case study of VR applications was developed using OVRInput and OVRHand Class Reference on Unity3D to differentiate virtual training practices [1]. We separated VR intubation training into two cases: VR controller and VR hand tracking. The experiments were designed on the same procedures, but the interactions were different. The VR controller was a virtual intubation training using the controller for interaction, while the VR hand tracking was also a virtual intubation training using hand gestures for interaction.

There are three main interactions in the VR application.

**Collision**

Interaction with virtual patients such as mouth opening can be done directly through the virtual hand of both VR controller and VR hand tracking where collision setting was applied. The user can touch and move the body parts of a virtual patient using a virtual hand.

**Pick up and release**

The user can pick up and place the virtual object by using LHandTrigger or RHandTrigger button on the VR controller and using a grab gesture on VR hand tracking.

**Squeeze and release**

The user can squeeze the Ambulatory bag by using LIndexTrigger or RIndexTrigger button on the VR controller and using a press gesture on VR hand tracking.

The virtual intubation training was divided into 6 procedures, including the introduction of tools and equipment. The sequence of endotracheal intubation was reviewed in a previous publication, the details were shown as follows:

**Introduction**

The equipment tray of endotracheal intubation (Fig. 1) such as the ambulatory bag, face mask, stethoscope, laryngoscope, endotracheal tube, stylet, oropharyngeal airway, syringe for inflating the cuff, lubricant gel, adhesive tape, and gloves [2,3].

**Procedure 1**

Proper sniffing position before laryngoscopy and intubation can help facilitate intubation by flexing the patient’s neck on the chest with a cushion under the occiput and extending [4].

**Procedure 2**

Pre-oxygenation with 100% oxygen (Fig. 2) and spontaneous ventilation with a tightfitting face mask connected with an ambulatory bag for 3–5 minutes before intubation. The right hand of the medical student controls the ambulatory bag while the left hand does control the face.

**Procedure 3**

Insert the laryngoscope by using the appropriate size of blade in the oral cavity and elevated the patient’s mandible. The medical students can see the epiglottis in this process [5].

**Procedure 4**

Lubricate the endotracheal tube with the lubricant gel then passing the endotracheal tube to the epiglottis, finally the endotracheal tube place in the trachea then removing the laryngoscope and inflating the cuff (Fig. 3).

**Procedure 5**

Connect the endotracheal tube with the ambulatory bag then ventilating the patient by using the right hand to squeeze the 1/3 of the ambulatory bag and using the left hand to control the endotracheal tube or connect the ventilation machine.

**Procedure 6**

Verify that the tube position is in the proper location (Fig. 4) to ventilate the patient’s lungs.

**References**

1. Map controllers [Internet]. Menlo Park (CA): Oculus.com [cited 2021 Jun 30]. Available from: https://developer.oculus.com/documentation/unity/unity-ovrinput.

2. Alanazi A. Intubations and airway management: an overview of Hassles through third millennium. J Emerg Trauma Shock 2015;8:99-107. https://doi.org/10.4103/0974-2700.145401

3. Bahathiq AO, Abdelmontaleb TH, Newigy MK. Learning and performance of endotracheal intubation by paramedical students: comparison of GlideScope(R) and intubating laryngeal mask airway with direct laryngoscopy in manikins. Indian J Anaesth 2016;60:337-342. https://doi.org/10.4103/0019-5049.181595

4. Gudivada KK, Jonnavithula N, Pasupuleti SL, Apparasu CP, Ayya SS, Ramachandran G. Comparison of ease of intubation in sniffing position and further neck flexion. J Anaesthesiol Clin Pharmacol 2017;33:342-347. https://doi.org/10.4103/joacp.JOACP_100_16

5. Duke JC, Keech BM. Duke’s anesthesia secrets. 5th ed. St Louis (MO): Saunders; 2016.
