# Supplementary material for: No difference in learning outcomes and usability between using controllers and hand tracking during a virtual reality endotracheal intubation training for medical students in Thailand
Source: J Educ Eval Health Prof. 2021 Aug 18;18:22. doi: 10.3352/jeehp.2021.18.22 (PMC8616725; doi:10.3352/jeehp.2021.18.22)
Supplement: Supplementary file 7 — Supplement 2. Intubation video. [file jeehp-18-22-suppl2.docx]

**Supplement 2.** Pre- and post-tests

**Pre-test and post-test for the knowledge test of the intubation**

1. Which choice is correct about the Stylet device during intubation?

a) Stylet is used to lubricate the mouth area and the tracheal tube.

b) Stylet is used to lubricate the hands and breathing tubes.

c) Stylet is used as the axis of arc-bending intubation.

d) Stylet is used to determine the depth of intubation by inserting it into the tracheal tube.

e) Stylet is used to determine if an endotracheal tube is inserted into the lungs.

2. Which choice is a device used for pre-oxygenation?

a) Laryngoscope

b) Ambulatory bag

c) Ventilator

d) Laryngoscope

e) Endotracheal tube

3. Which choice is the first step of intubation?

a) Pre-oxygenation

b) Placing the patient in the sniff position

c) Listening to both lungs of the patient

d) Ventilator connection

e) Examination of the endotracheal tube depth

4. Which choice is the last step of intubation?

a) Pre-oxygenation

b) Placing the patient in the sniff position

c) Lubricant the endotracheal tube

d) Ventilator connection

e) Examination of the endotracheal tube depth

5. Which choice refers to the pre-oxygenation process?

a) Giving 60% oxygen by using a face mask and ambulatory bag for 3–5 minutes.

b) Giving 80% oxygen by using a face mask and ambulatory bag for 3–5 minutes.

c) Giving 80% oxygen by using a face mask and ambulatory bag for 10–15 minutes.

d) Giving 100% oxygen by using a face mask and ambulatory bag for 3–5 minutes.

e) Giving 100% oxygen by using a face mask and ambulatory bag for 10–15 minutes.

6. Which choice is the correct order of the procedure for inserting the laryngoscope?

a) The laryngoscope is inserted after the lung auscultation procedure.

b) The laryngoscope is inserted after the face mask procedure to provide oxygen.

c) The laryngoscope is inserted after the ventilator installation procedure.

d) The laryngoscope is inserted before the patient positioning procedure.

e) The laryngoscope is inserted before the lubricate gel procedure.

7. Which choice is the correct order of the procedure for squeezing the ambulatory bag?

a) Squeezing the ambulatory bag is done after stylet insertion.

b) Squeezing the ambulatory bag is done after the procedure to cover the face mask.

c) Compression of the ambulatory bag is performed after the auscultation procedure.

d) Squeezing the ambulatory bag is performed after the patient positioning procedure.

e) Squeezing the ambulatory bag is performed after the ventilator connection procedure.

8. Which choice is the correct order of the procedure for inserting air into the cuff of the tracheal tube or the inflate cuff procedure?

a) The inflate cuff procedure is performed before the ventilator connection procedure.

b) The inflate cuff process is performed before the sniff position step.

c) The inflate cuff procedure is performed before the lubricating gel is applied.

d) The inflate cuff procedure is performed before the laryngoscope insertion procedure.

e) The inflate cuff process is performed before pre-oxygenation.

9. Which statement is correct about squeezing the ambulatory bag process?

a) Use your right hand to squeeze 1/2 of the ambulatory bag.

b) Use your right hand to squeeze 1/3 of the ambulatory bag.

c) Use your right hand to squeeze 1/4 of the ambulatory bag.

d) Use your right hand to squeeze 2/3 of the ambulatory bag.

e) Use your right hand to squeeze 3/4 of the ambulatory bag.

10. Which choice is the most correct sequence of steps for the auscultation of both lungs in the intubation?

a) Auscultation of both lungs is performed after endotracheal tube insertion.

b) Auscultation of both lungs is performed after the patient positioning procedure.

c) Auscultation of both lungs is performed after the stylet insertion procedure.

d) Auscultation of both lungs is performed after the pre-oxygenation.

e) Auscultation of both lungs is performed after the laryngoscope insertion.
