# Supplementary material for: No difference in learning outcomes and usability between using controllers and hand tracking during a virtual reality endotracheal intubation training for medical students in Thailand
Source: J Educ Eval Health Prof. 2021 Aug 18;18:22. doi: 10.3352/jeehp.2021.18.22 (PMC8616725; doi:10.3352/jeehp.2021.18.22)
Supplement: Supplementary file 11 — Supplement 6. Full questionnaire distributed to the cardiology residents. [file jeehp-18-22-suppl6.docx]

**Supplement 6.** Practice evaluations form for experts to assess the practice

**Evaluation form for experts to assess the practice**

| **No.** | **Criteria** | **Score (skip=0, incomplete=1, complete=2)** |
| --- | --- | --- |
| 1 | The sniff position of the patient is appropriate and correct. |  |
| 2 | Use the ambulatory bag to oxygenation the patient with the correct posture. |  |
| 3 | Open the patient’s mouth with the right hand, insert the laryngoscope into the corner of the mouth. |  |
| 4 | The endotracheal tube is inserted through the right corner of the mouth through the vocal cords into the trachea. |  |
| 5 | Remove the laryngoscope, use a syringe to insert air into the cuff, remove the stylet correctly |  |
| 6 | Connect the ventilator correctly. |  |
| 7 | Confirm the position of the end of the endotracheal tube correctly. |  |
|  | **Total (14)** |  |
